# Optimizing intra-facility crowding in Wi-Fi environments using continuous-time Markov chains

**DOI:** 10.1007/s43926-022-00026-x

**Published:** 2022-09-12

**Authors:** Shinya Mizuno, Haruka Ohba

**Affiliations:** Department of Computer Science, Shizuoka Institute of Science and Technology, 2200-2, Toyosawa, Fukuroi, Shizuoka 437-8555 Japan

**Keywords:** Optimization, Markov chain, Wi-Fi log, Parallel computing, K-means, Clustering, Facility operators, Crowdedness

## Abstract

Various measures have been devised to reduce crowdedness and alleviate the transmission of COVID-19. In this study, we propose a method for reducing intra-facility crowdedness based on the usage of Wi-Fi networks. We analyze Wi-Fi logs generated continually in vast quantities in the ever-expanding wireless network environment to calculate the transition probabilities between the nodes and the mean stay time at each node. Subsequently, we model this data as a continuous-time Markov chain to determine the variance of the stationary distribution, which is used as a metric of intra-facility crowdedness. Therefore, we solved the optimization problem by using stay rate as a parameter and developed a numerical solution to minimize the intra-facility crowdedness. The optimization results demonstrate that the intra-facility crowding is reduced by approximately 30%. This solution can practically reduce intra-facility crowdedness as it adjusts people’s stay times without making any changes to their movements. We categorized Wi-Fi users into a set of classes using the k-means method and documented the behavioral characteristics of each class to help implement class-specific measures to reduce intra-facility crowdedness, thus enabling facility managers to implement effective countermeasures against crowdedness based on the circumstances. We present a detailed description of our computing environment and workflow used for the basic analysis of vast quantities of Wi-Fi logs. We believe this research will be useful for analysts and facility operators because we have used general-purpose data for analysis.

## Introduction

The COVID-19 pandemic has forced governments, municipalities, and other institutions worldwide to implement strict countermeasures against the transmission of the disease. In addition to hygiene countermeasures, such as mouth rinsing and hand washing, it is important to stop crowd gatherings [1]. The increased use of online platforms has helped alleviate this issue but has resulted in changes to the existing physical environments, thus facilitating the requirement for objective indicators to determine the crowdedness of a given facility.

Measuring crowdedness is important for developing COVID-19 countermeasures and optimizing crowdedness to help overcome imbalances in the number of customers congregating in a certain area of interest. In the transportation field, the effects of crowdedness have been documented from the perspectives of accessibility and mobility [2]. Cameras have been increasingly used to analyze the movements of people in buses and other similar settings in recent years [3, 4]. However, it is difficult to analyze simultaneous events at multiple points since cameras capture only a specific point in space.

Global internet usage reached approximately 63% in 2021, making it an indispensable part of our daily lives [5]. The increasing usage of smartphones worldwide is primarily attributed to the increased internet connectivity over both mobile networks and Wi-Fi. The Wi-Fi environment presents various applications and is used in education, tourism, and disaster management and prevention, leading to the establishment of national Wi-Fi policies in several countries across the world. The Wi-Fi environment is the most preferred network environment owing to the advent of 5G and other technologies and is expected to be used widely in virtual reality, remote offices, online classes, and various other fields, such as smart homes, support for the elderly, and nursing care [6–9].

In this study, we propose an optimization method to avoid crowding in facilities, thereby limiting the transmission of COVID-19. We analyze Wi-Fi logs created regularly across different Wi-Fi environments to determine the behavior of the users. In the proposed method, the constraints set ensure that users and facility managers can take realistic steps against crowdedness without drastically changing the status quo. Firstly, we used Wi-Fi log statistics to perform a basic analysis of Wi-Fi users. We then classified users based on factors, such as frequency of use, location, and stay time, and identified the user groups present in the facility. Subsequently, we obtained information on the transition probabilities between nodes and the stay time at each node for each user group, which were modeled as a continuous-time Markov chain. If a stationary distribution was obtained, the variance of the stationary distribution was used as a metric to assess the intra-facility crowdedness. We then performed optimization under certain constraints, considering the stay time parameter of each node as the explanatory variable and the variance of the stationary distribution as the objective function. This approach can help in alleviating crowdedness in practical conditions. The proposed optimization method effectively utilizes Wi-Fi environment logs to help facility operators limit the transmission of COVID-19.

### Related work

Extensive research has been conducted on determining user behavior using Wi-Fi logs in various fields [10–21]. In particular, studies have been conducted on tourism behavior analysis using Wi-Fi logs to better understand the state of tourism behavior based on facility and visitor stay times and OD (Origin–Destination) Tables [22, 23]. For example, Internet-of-Things (IoT) data transmitted through Bluetooth and Wi-Fi have been used to monitor and estimate the number of passengers and the waiting time for buses and subway trains [24, 25]. Wi-Fi data have also been used to track tourists and determine the appeal of tourist attractions [26]. This Wi-Fi data tracking enables a strategic implementation of services, including estimating factors affecting tourism and presenting recommendations to tourists [27, 28]. However, these applications have not accelerated the use of Wi-Fi data for strategic planning thus far.

In previous studies, Markov chains were used to understand tourism behavior [29, 30]. These studies did not consider properties such as reachability or stationary distribution as they were based on the concept of absorbing Markov chains, wherein the user arrives from outside. Additionally, data-cleaning methods have been examined owing to the large volume of Wi-Fi log data [31]. However, the research conducted on the computing environment and the methods used to analyze large quantities of log data has been limited. The computing environment which handles the ever-increasing log data is significant for achieving real-time results. The Wi-Fi logs provided by vendor tools typically include basic information, such as the number of user connections, connecting devices, authentication status, and usage status. The arrival rate per unit time and the stay time of each user can be obtained. However, the vendor-supplied tools do not track user transitions through access points, which limits their ability to present a detailed overview of user trends.

Several efforts have been made to mitigate the impact of the COVID-19 pandemic, including investigating and predicting infection and movement of people [32, 33]. Ainslie et al. [34, 35] observed a strong correlation between intra-urban migration and the infection rate based on the intra-city movement data obtained during the early stages of the pandemic. In addition, susceptible, infected, and recovered (SIR) models have been used to investigate and predict the infections [9, 36–38].

IoT devices have also been used to monitor and intervene in public health in densely populated areas. A recent study incorporated machine learning approaches to maximize the testing resources to track people that have come into contact with an infected person [39–41]. Wang et al. [42] observed that interventions focusing on highly-mobile individuals and popular locations rather than the movements of actual people captured by Wi-Fi and GPS could reduce both peak infection rates and the total number of infected people while maintaining high social activity levels. However, none of these studies have attempted the optimization of crowdedness.

In this study, we have developed an environment wherein Wi-Fi logs can be analyzed realistically, and we propose a method to define and optimize intra-facility crowdedness. This approach enables the implementation of realistic anti-crowding measures and addresses the issues surrounding the global COVID-19 pandemic.

### Features of this study

This study focuses on the environment along with analyzing large amounts of Wi-Fi logs and calculating their statistics. Table 1 presents a comparison of the findings of this study with that of a previous study. The left side of the table presents items related to the scale of the Wi-Fi logs, while the right side presents items related to the analysis. In addition to basic statistical methods, clustering of users is also important to analyze the logs, which present a better understanding of the trend of Wi-Fi users. Further optimization can help in the development of an improved environment. This study utilizes a sufficient amount of Wi-Fi logs for analysis, along with a log calculation flow and large-scale computing environment. The proposed analysis method presents considerable academic and practical significance as it enables system optimization through statistical analysis and user classification.

**Table 1:** Comparison between the findings of this study and that of previous studies

Reference numbers	Number of APs	Number of users (devices)	Number of data	Log computing environment and Algorithm	Statistical analysis	Clustering	Optimization
This study	1123	33,837	7,655,584	Mentioned	Mentioned	Mentioned	Mentioned
[11]	NA	NA	NA	NA	Mentioned	NA	NA
[13] US	Approx. 5,500	Approx. 38,000	NA	Mentioned	Mentioned	Mentioned	NA
[13] Singapore	Approx. 13,000	Approx. 50,000					
[14]	550	5989	1,437,504	NA	Mentioned	NA	NA
[15]	NA	123	43,150	NA	Mentioned	Mentioned	NA
[16]	37	7336	NA	NA	Mentioned	NA	NA
[17]	28	27,538	300,681	NA	Mentioned	Mentioned	NA
[18]	8	69,467	4,517,687	NA	Mentioned	NA	NA
[19]	11,964	Approx. 30,000	216 million	NA	Mentioned	Mentioned	NA

## Materials and methods

In this study, we used Wi-Fi logs to optimize the intra-facility crowdedness. This section details the proposed approach for processing large quantities of Wi-Fi logs and the computing environment needed. We calculated the characteristics of different users and organized them into groups using the characteristic quantities obtained from the processed Wi-Fi logs. We then used these user groups to assess intra-facility crowdedness based on a continuous-time-type Markov chain. Lastly, we developed an optimization model which minimizes the intra-facility crowdedness. The optimization model imposes a fixed limit on the variability of the stay time by using the stay time of the user as a parameter, making it a realistic solution. Congestion in a facility can be avoided using two methods: (1) changing the flow lines within the facility, and (2) changing the time spent within the facility. However, method (1) requires major changes to both the customers and the facility. However, method (2) requires changes only to the length of stay, and the facility remains largely unaffected. Furthermore, in the absence of a constraint to reduce congestion, the facility would simply reduce it, thereby reducing the value of the facility. Thus, we could match the actual conditions at the facility by using method (2) and setting additional constraints.

### Process from Wi-Fi log to transition probability matrix calculation

#### Items used in the Wi-Fi logs

The WPA2-Enterprise with 802.1X authentication and WPA2-PSK (shared network key) were used as the main security nodes for a basic authentication at a Wi-Fi access point. These authentication methods require the following log items collected by most logging programs and used in this analysis, i.e., connection time, destination access point, and unique user identifier.

#### Overview of the Wi-Fi log data used

We used the Crawdad.org dataset for our Wi-Fi log data, which is a dataset of association records for the Eduroam network at the KTH campuses, collected during 2014–2015 [43]. Table 2 lists access point (AP) information for this dataset, including AP name and location information. A total of 1,123 APs were listed in the database.

**Table 2:** Excerpt from APlocations.csv (1,123 items in total)

AP	x_coordinate (m)	y_coordinate (m)	Floor
Bldg1AP1	21,534	32,313	2
Bldg1AP2	21,534	32,313	2
Bldg1AP3	21,534	32,313	3

Table 3 lists the content of the file containing user connection information, which includes a unique user identifier, the AP to which the user is connected, and the connection time. Table 4 lists the files in the dataset organized on a monthly basis. The data containing N/A and users with only one connection were excluded. We targeted users who had connected more than once and had transitions between APs.

**Table 3:** Excerpt from traceset/2014_01.csv

Timestamp	Client	AP
2014-01-01 00:00:24	b7f22f…	Bldg11AP21
2014-01-01 00:00:30	336501…	Bldg44AP3
2014-01-01 00:00:35	4b912f…	Bldg48AP65

**Table 4:** Summary of monthly usage

Year/month	Total number of records	Number of users	Amount of data (Mbyte)
2014/01	5,634,970	27,052	402.5
2014/02	6,803,432	27,164	485.9
2014/03	6,671,451	28,351	476.4
2014/04	6,178,580	28,178	441.3
2014/05	6,466,906	30,525	461.8
2014/06	3,284,709	21,776	234.8
2014/07	1,426,694	8495	102.1
2014/08	3,605,161	18,787	257.5
2014/09	7,655,584	33,837	546.2
2014/10	7,401,092	34,374	528.2
2014/11	7,114,147	34,107	507.6
2014/12	5,366,380	31,738	383.0
2015/01	5,813,530	32,057	414.8
2015/02	6,806,318	32,428	485.5
2015/03	7,235,589	33,750	516.2
2015/04	5,818,219	32,417	415.2

#### Calculation of transition probabilities and stay times

The process for calculating the transition probabilities and stay times from the Wi-Fi logs is presented below. This process is a general-purpose workflow for WiFi logs.

A. Preprocessing for Wi-Fi log analysis:
  1. Delete records containing “N/A” from the original file.
  2. Delete users who appear only once in the original file.
  3. Output the files processed in (1) and (2).
B. Main processing for Wi-Fi log analysis:
  1. Calculate the transition probabilities through parallel computation by using the Message Passing Interface (MPI), based on the file created in A (preprocessing).
  2. Each MPI process calculates the number of transitions and stay times of the specified user and outputs the results to a file with the aggregated information of all the users.
C. Post-processing for Wi-Fi log analysis:
  1. Delete APs with no transitions and calculate the transition probability matrix from the file obtained in B (main processing).
  2. Calculate the transition probability matrix and mean stay time for each classroom, floor, and building with the APs aggregated and output the results to a file.

**Fig. 1:**
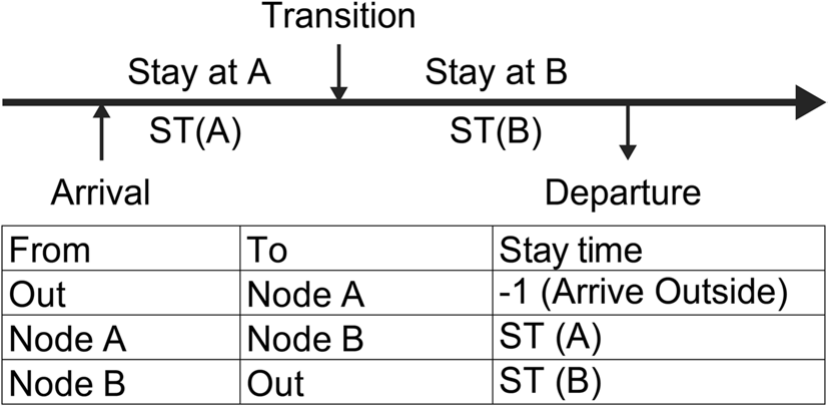
State transition in Wi-Fi logs

Figure 1 illustrates the state transition obtained in Section B. (Main process for calculating transition probabilities).

#### Process for calculating user groups

We performed clustering using the k-means method to analyze the characteristics of Wi-Fi users. We created a dataset for each user based on calculation results of transition probabilities and stay times in the format presented below. Here, the node number indicates the number assigned when the AP aggregation is performed. Table 5 presents the information on the environment in which clustering was computed.

**Table 5:** Computing environment for user clustering

Item	Description
Programming language	Python 3.7.12
Library used	scikit-learn = 1.0.1
Technique	K-Means
Number of clusters	5

User *i* = {node 1: number of connections …, node N: number of connections, node 1: stay time …, node N: stay time}.

### Computing environment for Wi-Fi log analysis

The Wi-Fi logs are enormous, and because the number of access points and users increases, the computation time increases. Therefore, a simple computing environment cannot adequately handle the seamless operation of businesses. Consequently, we used a parallel computing environment to improve the efficiency of the Wi-Fi log computations. Table 6 lists the information on the programming environment used in this study. Additionally, we used the SQUID computing environment [44] at the Cybermedia Center of Osaka University. The file for 2014/09, which possessed the largest number of records, required a computation time of 8836.68 s. The computation time can be further reduced by increasing the amount of parallelism based on the availability of computing resources.

**Table 6:** Computing environment for Wi-Fi log analysis

Item	Description
Programming language	Python 3.6.13
Library used	mpi4py 3.1.3
Computing environment	SQUID (Osaka University)
Processor information	Intel(R) Xeon(R) Platinum 8368 Packages(sockets): 2 Cores: 76 Processors (CPUs): 152 Cores per package: 38 Threads per core: 2
Memory	248 GB/Node
Number of nodes used	1
Number of cores used	76 (all in parallel)

### Measuring intra-facility crowdedness from Wi-Fi logs using continuous-time Markov chains

In this section, we first define the facility crowdedness using a continuous-time Markov chain [45]. We assume a finite state space, $S$, and continuous-time stochastic process, $\{X\left(t\right);t\ge 0\}$. We define a continuous-time Markov chain with the transition probability, ${\varvec{P}}\left(t\right)= \left({p}_{ij}(t)\right), i,j\in S$ on $S$. Here, $X(t)$ is assumed to satisfy the following equation and be synchronous:

$${p}_{ij}(t)= P\left(X\left(s+t\right)=j|X\left(s\right)=i\right), i,j\in S$$

In addition, for each transition probability, ${p}_{ij}(t)$,

$${q}_{i}=\underset{h\downarrow 0}{\mathit{lim}}\frac{1-{p}_{ii}(h)}{h}\in \left[0, \infty \right], i\in S,$$

$${q}_{ij}=\underset{h\downarrow 0}{\mathrm{lim}}\frac{{p}_{ij}\left(h\right)}{h}\in \left[0,\infty \right], i\in S, i\ne j.$$

We define the transition rate matrix assuming ${q}_{ii}=-{q}_{i}, i\in S$, as follows:

$${\varvec{Q}}={(q}_{ij})=\underset{h\downarrow 0}{\mathrm{lim}}\frac{\left\{{\varvec{P}}\left(h\right)-{\varvec{I}}\right\}}{h},$$

where $${\varvec{P}}\left(0\right)={\varvec{I}}$$.

At time, $t$, when $X(t) = i$ and $i\in S$, the probability that the remaining stay time, ${\tau }_{i}(t)$, is greater than $u$ is given by:

$$P\left({\tau }_{i}\left(t\right)>u|X\left(t-\right)\ne i,X\left(t\right)=i\right)={e}^{-{a}_{i}u},$$

where $\frac{1}{{a}_{i}}$ represents the mean stay time for $i\in S$. Therefore, the transition rate, ${q}_{ij}$, can be expressed as:

1 $$\begin{array}{c}{{q}}_{{i}{j}}=\left\{\begin{array}{c}-{{a}}_{{i}} \left({i}={j}\right)\\ {{a}}_{{i}}{{p}}_{{i}{j}} \left({i}\ne {j}\right)\end{array}\right.\end{array}$$

When $X(t)$ is irreducible and ergodic, there is a limit distribution for $j\in S$

$${\pi }_{j}=\underset{t\to \infty }{\mathrm{lim}}{p}_{ij}\left(t\right)\ge 0, {\sum }_{j\in S}{\pi }_{j}=1,$$

where $${\pi }_{j}$$ satisfies:

2 $$\begin{array}{c}{\sum }_{{i}\in {S}}{{\pi}}_{{i}}{{q}}_{{i}{j}}=0,\boldsymbol{ }\left({j}\in {S}\right)\end{array}$$

and ${\pi }_{i} (i\in S)$ denotes the stationary distribution.

To define intra-facility crowdedness, we consider $\sigma$ as the variance of the stationary distribution of each state. Therefore,

$$\sigma \left({\varvec{a}}\right)=\frac{1}{\left|S\right|}{\sum }_{i\in S}{{(\pi }_{i}-\overline{\pi })}^{2},$$

where $$\overline{\pi }=\frac{1}{|s|}{\sum }_{i\in S}{\pi }_{i}$$.

### Method for optimizing intra-facility crowdedness

Subsequently, we propose a method to optimize the crowdedness of the facilities [46]. In this section, we classify the users based on their usage and introduce a set, $C$, of the user classes. Intra-facility crowdedness is defined as the variance of the stationary distribution of each state. Therefore, we can reduce the intra-facility crowdedness by minimizing this variance, as shown in Eq. (3). Here, the sum of the mean stay times in the facility is set as a constant value for each user class to avoid any hindrances in the usage of the facility, as shown in Eq. (4). Here, ${{\varvec{a}}}^{\boldsymbol{^{\prime}}({c})}$ denotes the initial value of ${{\varvec{a}}}^{({c})}$, ${\pi }_{i}^{\left(c\right)}\left({a}_{i}^{\left(c\right)}\right)$ denotes the stationary distribution, given the stay rate, ${a}_{i}^{(c)}$, node, $i$, and user class, $c$, and $\overline{\pi }\left({a}_{i}^{\left(c\right)}\right)$ denotes the mean value of the stationary distribution. A drastic change in the stay rate of users may cause confusion; therefore, we limit the change to a certain range for each user class, as shown in Eq. (5).

3 $$\begin{array}{c}Minimize \quad \sigma \left({{\varvec{a}}}^{\left(1\right)},\dots ,\boldsymbol{ }{{\varvec{a}}}^{\left({c}\right)},\dots \right)=\frac{1}{\left|{S}\right|}\sum_{{c}\in {C}}\sum_{{i}\in {S}}{\left({{\pi}}_{{i}}^{\left({c}\right)}\left({{a}}_{{i}}^{\left({c}\right)}\right)-\boldsymbol{ }\overline{{\pi} }\left({{a}}_{{i}}^{\left({c}\right)}\right)\right)}^{2}\end{array}$$

$$Subject \quad to \quad {a}_{i}^{(c)}\ge 0, i\in S, c\in C$$

4 $${\sum }_{i\in S}\frac{1}{{a}_{i}^{(c)}}={K}^{(c)}, {K}^{(c)}\in {\mathbb{R}}$$

5 $${{\varvec{a}}\boldsymbol{^{\prime}}}^{(c)}\left(1-{\gamma }^{(c)}\right)\le {{\varvec{a}}}^{\left({c}\right)}\le {{\varvec{a}}\boldsymbol{^{\prime}}}^{\left(c\right)}\left(1+{\gamma }^{\left(c\right)}\right), {\gamma }^{\left(c\right)}\in {\mathbb{R}}$$

**Facility crowdedness optimization algorithm**

The facility crowdedness optimization algorithm is as follows:

1. Classify users into classes$,$ $C,$ based on facility Wi-Fi logs.
2. For each class, $c\in C$, $calculate$ the transition probability matrix, ${{\varvec{P}}}^{c}\left(t\right)=\left({p}_{ij}^{c}\left(t\right)\right), i,j\in S, c\in C$, and set the initial value of the stay time parameter, ${a}_{i}^{(c)}$, based on the flow described in 2.1.3.
3. Create the transition rate matrix, ${{\varvec{Q}}}^{(c)}$, based on Equation ($1$) and obtain ${\pi }_{i}^{(c)}$ from Equation ($2$).
4. Solve the optimization problem ($3$) and determine an ${{\varvec{a}}}^{({c})}$ value which satisfies the conditions.
5. Repeat steps (3) and (4) to calculate the optimal ${{\varvec{a}}}^{({c})}$ value.

Table 7 presents the information on our computing environment used for optimizing the intra-facility crowdedness.

**Table 7:** Environment for optimizing intra-facility crowdedness

Item	Description
Programming language	Python 3.7.12
Library used	scipy = 1.4.1
Optimization algorithm	Constrained trust region method
Explanatory variable	Stay rate ${a}^{(c)}$ at each node
Gradient function	Gradient estimation (2-point) applied
Hessian matrix	Hessian estimation (BFGS) applied
${\gamma }^{(c)}$	${\gamma }^{(c)}=0.25$ in all classes

## Results and discussion

### Basic user analysis obtained from Wi-Fi logs

**Fig. 2:**
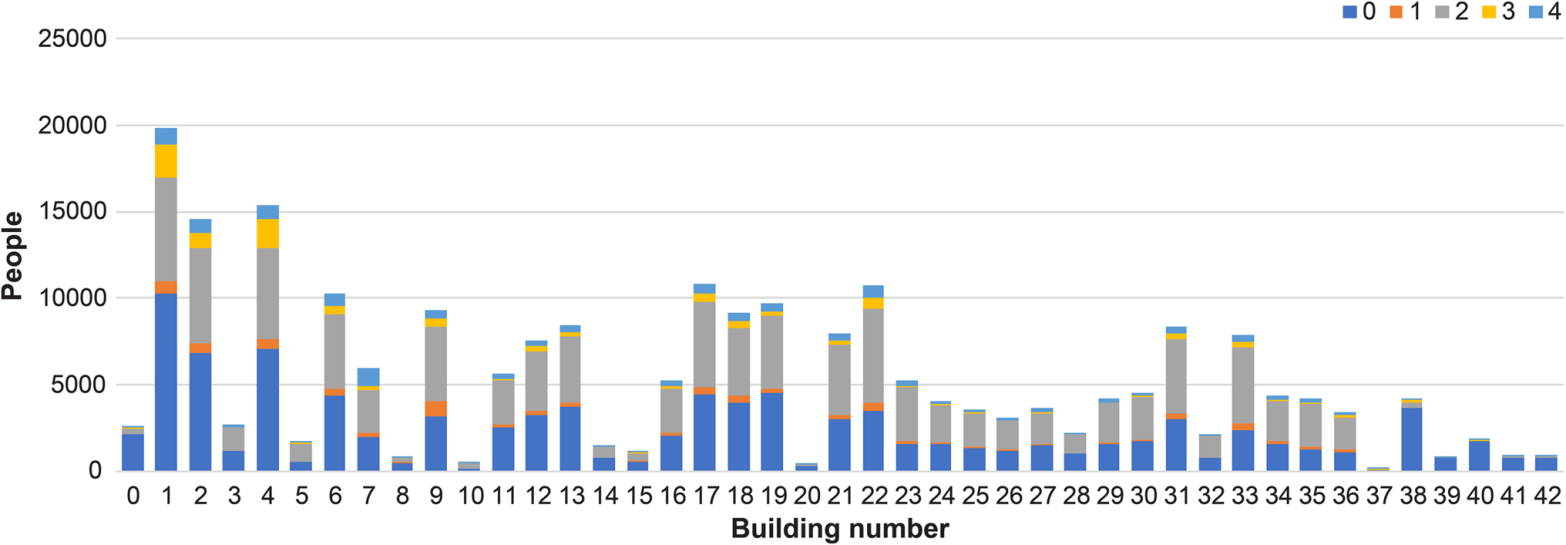
Number of users who used the Wi-Fi in each building node at least once

We categorized the users into five classes based on the number of connections, location, and stay time. Table 8 lists the characteristics of each class. Figure 2 depicts the number of users who used Wi-Fi in each building at least once. Most users belonged to Class 0 and had a short mean stay time. These could be outside users or students who do not regularly use the network as the data used in this study were obtained from Eduroam, which is a Wi-Fi roaming service that allows access from outside the university where it is installed.

**Table 8:** Characteristics of each class

User class	Number of users	Mean stay time (h)	Number of building nodes			
			1/3 of cluster stays	Mean stay time < 1 h	Mean stay time < 2 h, $\ge$ 1 h	Mean stay time < 3 h, $\ge$ 2 h
0	23,221	0.003	1	25	14	4
1	879	0.442	10	18	5	20
2	6476	0.050	22	12	5	26
3	2225	0.092	3	22	9	12
4	1036	0.345	15	15	6	22

We clustered the buildings by using the mean stay times of the users in each building (mean stay time for user class 0, mean stay time for user class 1,…, mean stay time for user class 4) to obtain an accurate classification of the characteristics of the user classes. Table 9 presents the results. Except for user class 0, more than 75% of all the user classes were present in building cluster 2 at least once. The table presents the characteristics of each user class, e.g., user class 2 uses building cluster 4, user class 3 uses building cluster 2, and so on.

**Table 9:** Clustering of building nodes

Building cluster	0		1		2		3		4	
Building number	6, 7, 12, 13, 17, 18, 19, 21, 31, 33		0, 5, 8, 10, 14, 15, 20, 28, 32, 37, 38, 39, 40, 41, 42		1, 4		3, 11, 16, 23, 24, 25, 26, 27, 29, 30, 34, 35, 36		2, 9, 22	
User class	rate (%)	time (h)	rate (%)	time (h)	rate (%)	time (h)	rate (%)	time (h)	rate (%)	time (h)
0	14.9	0.023	4.2	0.011	37.3	0.054	6.7	0.011	19.4	0.031
1	35.9	0.147	2.7	0.008	75.8	0.291	12.7	0.038	72.7	0.66
2	61.7	0.161	6.1	0.016	86.3	0.179	33.5	0.065	78.6	0.275
3	14.3	0.051	1.4	0.006	81.6	0.691	4.1	0.013	29.3	0.105
4	50.3	0.233	3.3	0.008	84.8	0.266	20.4	0.044	63.7	0.24

### Evaluation of intra-facility crowdedness by class

We evaluated the intra-facility crowding in each class. Table 10 presents the class-specific values related to intra-facility crowding. The mean stay time (h) was the mean value of stay time in each building for each class. The total mean stay time (h), which is the sum of stay times in each building, is a constraint imposed on the optimization. The intra-facility crowdedness was computed for each class. Class 3 presents the largest mean stay time, the largest sum thereof, and the greatest intra-facility crowdedness.

**Table 10:** Class-specific values related to intra-facility crowdedness

Class ($c$)	Mean stay time (hours) ($Mean({a}^{\left(c\right)})$)	Total mean stay time (hours) (${K}^{(c)}$)	Intra-facility crowdedness
Class 0	0.5056	21.74	0.000813
Class 1	0.8170	35.13	0.001513
Class 2	0.6227	26.77	0.001223
Class 3	0.8276	35.58	0.002853
Class 4	0.6617	28.45	0.001469

**Fig. 3:**
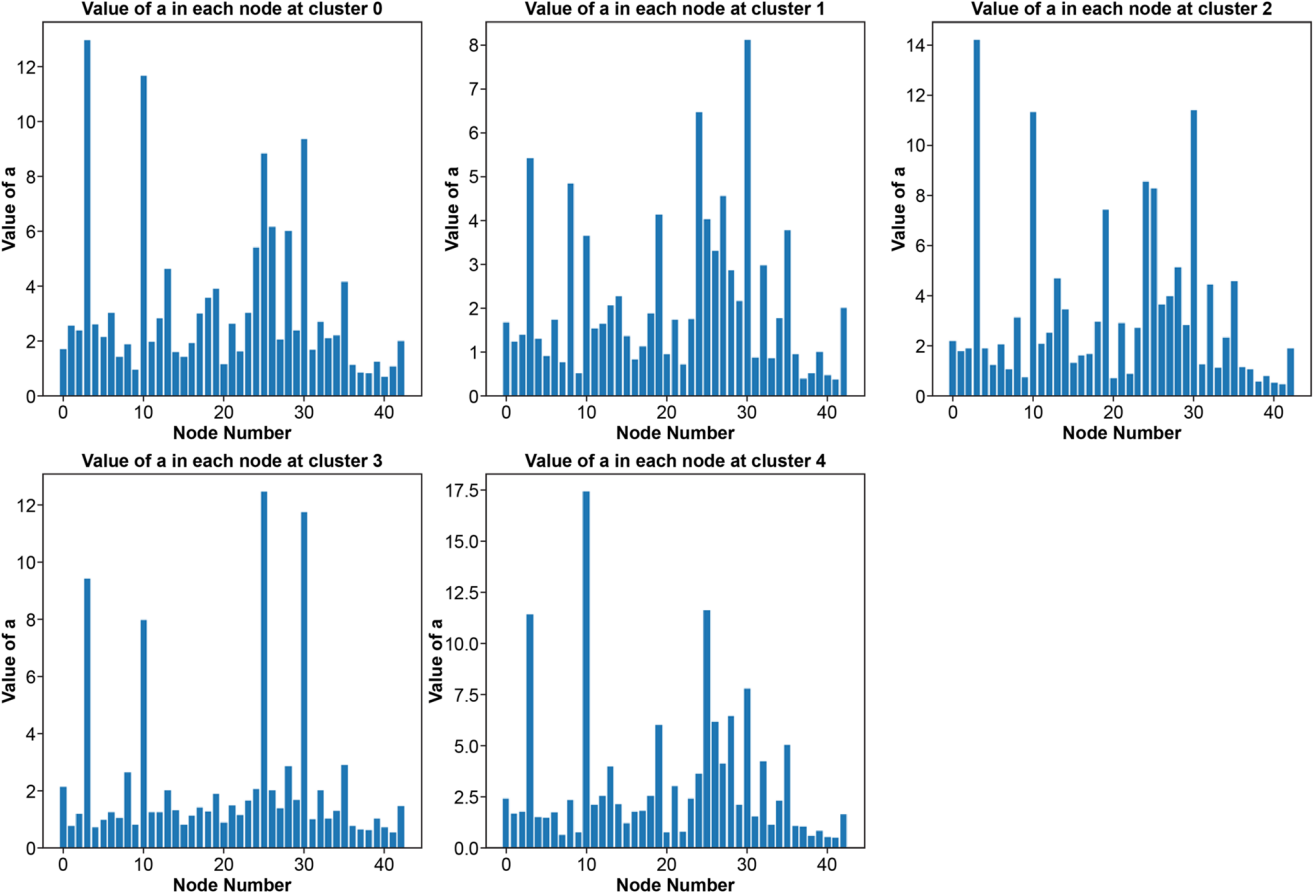
Stay rate for each class

**Fig. 4:**
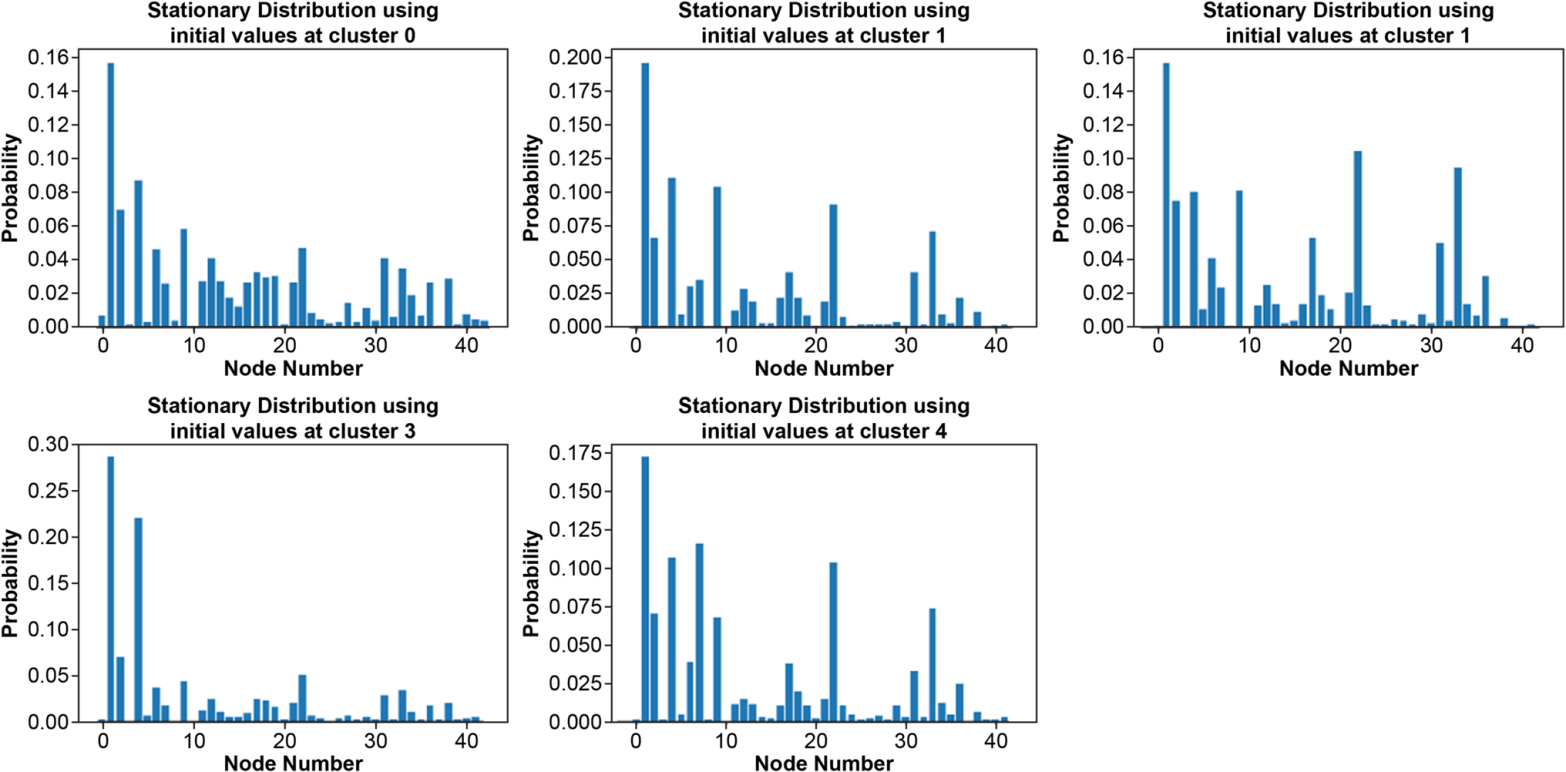
Stationary distribution for each class

Figures 3 and 4 depict the stay rate and stationary distribution for each class, respectively.

### Implementation of intra-facility crowdedness optimization

The pre-optimized intra-facility crowding for all users was 0.00157494. Table 6 presents the information on the optimization computing environment. The number of iterations was 1502, and the computation time was 3196.20 s. The intra-facility crowding after optimization was 0.00109697. The total mean stay time is a non-varying constraint; therefore, we checked the mean and standard deviation of the stay rate and the change in the intra-facility crowdedness in each class, as shown in Table 11. It can be observed that the optimization caused a decrease in the overall intra-facility crowdedness as well as the cluster-specific intra-facility crowdedness.

**Table 11:** Changes in stay rate and intra-facility crowdedness after optimization

Class ($c$)	Before optimization	After optimization	Before optimization	After optimization	Reduction rate (%)
	Mean and standard deviation of stay rate	Mean and standard deviation of stay rate	Intra-facility crowdedness	Intra-facility crowdedness	
Class 0	3.1941, 2.7725	2.9431, 2.6706	0.000813	0.000560	68.8806
Class 1	2.1562, 1.7184	2.1876, 1.9235	0.001513	0.001072	70.8526
Class 2	3.2556, 3.1473	2.8221, 2.7170	0.001223	0.000863	70.5641
Class 3	2.1756, 2.7388	2.4671, 3.4307	0.002853	0.001924	67.4377
Class 4	3.1551, 3.3967	2.7481, 3.1901	0.001469	0.001063	72.3621

**Fig. 5:**
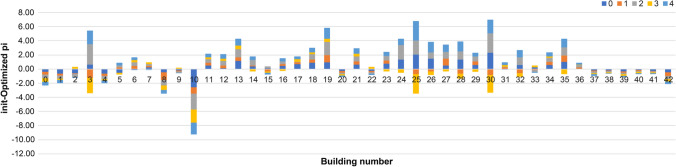
Change in stay rate in each class before and after optimization

**Fig. 6:**
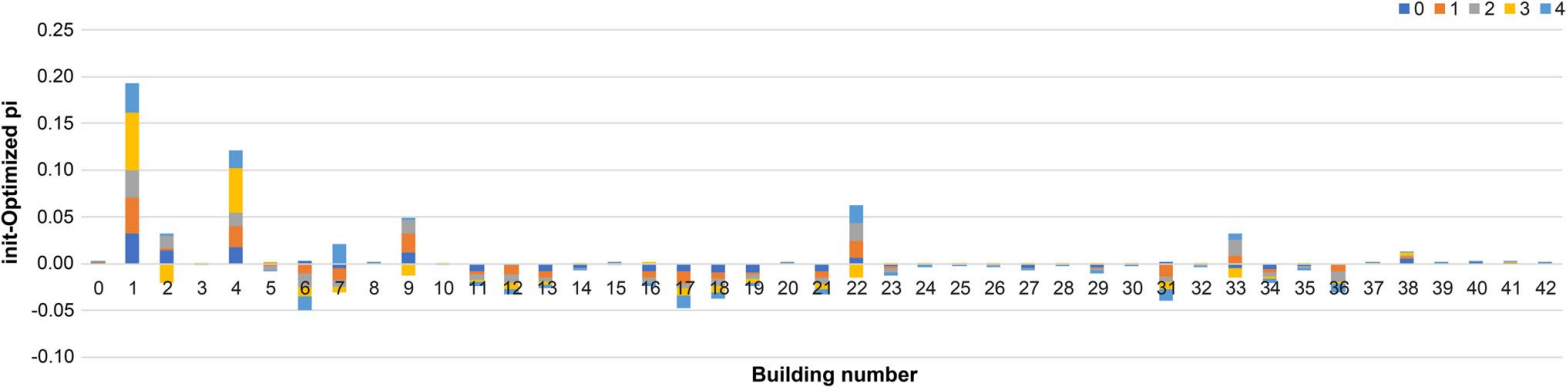
Change in stationary distribution for each class before and after optimization

Figure 5 depicts the change in the stay rate before and after optimization. The negative values indicate buildings where the stay rate increased. The stay rates for Building 1 decreased compared to the values in Figs. 3 and 4, indicating that the stay time is longer and the value of the stationary distribution is greater for all classes. The optimization increases the stay rate and reduces the stay time to standardize the value of the stationary distribution of this building, as shown in Fig. 5. Furthermore, it reduces the stationary distribution, as shown in Fig. 6. In Building 30, the stay rate was high, and the stationary distribution was low. After optimization, the stationary distribution could be increased by decreasing the stay rate in classes 0, 2, and 4 and increasing it in classes 1 and 3. Therefore, the increase or decrease in the stay rate, even for the same building, varies based on the class, demonstrating that this method can provide realistic optimization results.

## Conclusion

In this study, we presented a computational algorithm and its environment for effectively using huge Wi-Fi logs and classified the Wi-Fi users based on clustering. We also proposed an optimization model by applying the transition probability matrix and stay rate obtained from Wi-Fi logs to a continuous-time Markov chain. This optimization model can effectively prevent intra-facility crowding, as demonstrated through numerical calculations. The model can reduce the crowding in the facility without changing the transition probability matrix, i.e., without changing the flow line of people and only changing the stay rate. Additionally, the model can be easily adopted for facility management as the optimization can be performed for each user class. The proposed optimization model utilizes Wi-Fi logs to prevent user crowding and simultaneously increases the effectiveness of the facility operations while preventing the transmission of COVID-19.

The main limitation of this study is that there is no disconnection time available in the Wi-Fi logs. Therefore, we have set 3 h as the maximum time spent. If there is no Wi-Fi cut-off time, the accuracy can be improved by performing survival time analysis [47] on the time spent. The objective function of the optimization model is the variance of the stationary distribution. Ohsaki [46] uses an objective function that considers the facility area and the number of people the facility can accommodate. It is essential to compare this with an objective function that includes the structure of the facility.

## Data Availability

The data that support the findings of this study are available from the CRAWDAD project, but restrictions apply to the availability of these data, which were used under license for the current study, and are therefore not publicly available. However, the data can be provided by the authors upon reasonable request and with the permission of CRAWDAD. All the source code used for the calculations in the text is available at https://github.com/smzn/Optimizing-intra-facility-crowding-in-Wi-Fi-environments-using-continuous-time-Markov-chains.

## Abbreviations

AP: Access point
GPS: Global positioning system
IoT: Internet of things
MPI: Message passing interface
OD: Origin–destination
SIR: Susceptable, infected, and recovered model
